# Accelerated flat panel computed tomography for pre-operative temporal bone imaging: Image quality and dosimetry comparison to conventional high resolution multislice computed tomography

**DOI:** 10.1007/s00234-025-03592-3

**Published:** 2025-03-25

**Authors:** Elie Diamandis, Sebastian Johannes Müller, Eya Khadhraoui, Stefan Klebingat, Eric Einspänner, Martin Durisin, Anne Albrecht, Daniel Behme

**Affiliations:** 1 University Clinic for Neuroradiology, Otto-Von-Guericke-University Magdeburg, Magdeburg, Germany; 2 German Center for Neurodegenerative Diseases (DZNE), Magdeburg, Germany; 3 University Clinic of Otolaryngology, Head and Neck Surgery, Otto-Von-Guericke-University Magdeburg, Magdeburg, Germany; 4 Institute of Anatomy, Otto-Von-Guericke-University Magdeburg, Magdeburg, Germany

**Keywords:** Flat-panel computed tomography, Cone-beam computed tomography, Multislice computed tomography, Temporal bone imaging, Anatomy

## Abstract

**Purpose:**

High-resolution multislice CT (HR-MSCT) and cone beam CT (CBCT) are commonly used for preoperative temporal bone imaging, with HR-MSCT often preferred due to its shorter scan duration and lower susceptibility to motion artifacts. However, recent advancements in accelerated flat panel CT (Acc-FPCT) available with the latest generation angiography systems have addressed traditional limitations of CBCT by significantly decreasing scan time. This cadaver-based study evaluates the diagnostic performance and radiation dose of Acc-FPCT compared to HR-MSCT in preoperative temporal bone imaging.

**Methods:**

Six different Acc-FPCT protocols were acquired on five whole-head cadaveric specimens (ten temporal bones). Three neuroradiologists experienced in temporal bone imaging assessed the image quality of Acc-FPCT protocols in comparison to that of HR-MSCT for the visualization of 31 landmarks of middle and inner ear using a 5-point Likert scale. We also compared radiation dose parameters (CT dose index and dose length product) among the protocols.

**Results:**

Two high-Resolution Acc-FPCT protocols were found to be superior to HR-MSCT by all raters (*p* < 0.001). There were no significant differences between the two HR-FPCT protocols (*p* = 0.25). The remaining Acc-FPCT protocols were rated significantly inferior to HR-MSCT. The inter-rater reliability was excellent (ICC (2,k) = 0.925; CI [0.92–0.93]). The dose length product was significantly lower in all Acc-FPCT protocols compared to HR-MSCT.

**Conclusion:**

The results of our cadaver-based study highlight the utility of certain Acc-FPCT protocols as a viable alternative to HR-MSCT in preoperative temporal bone imaging, improving the visualization of critical anatomical landmarks without increasing radiation exposure.

## Introduction

Computed tomography (CT) is the cornerstone in perioperative imaging in patients with conductive and sensorineural hearing loss. It has the capacity to assess the integrity of the ossicular chain, identify congenital defects in bony labyrinth and internal auditory canal, estimate the cochlear duct length and accurately plan the insertion path of cochlear implant electrode [1–6].

Different CT scanning methods have been used in this setting with high-resolution multislice CT (HR-MSCT) being the mainstay modality. Shortly after its clinical introduction in the late 1990s, Cone beam CT (CBCT) has proven to be a viable alternative to MSCT in dental, maxillofacial and temporal bone imaging [7].

The single rotation volumetric acquisition of CBCT allowed for lower radiation dose in comparison to MSCT. In addition CBCT was extensively reported to be useful in ENT imaging providing superior spatial resolution, higher contrast and fewer metal-related artefacts [8–11]. Development of flat detector technology represented another milestone in CBCT imaging, allowing for increased spatial resolution and contrast compared to earlier intensifier detectors [12]. In the last two decades, major advancements in digital flat panel technology were achieved, mainly in detector size, pixel size, frame rate and bit-depth [13].

The availability C-arm mounted flat-panel CT (FPCT) scanners as an integral part of modern interventional suites led to a wider use of FPCT in diagnostic imaging [14]. In many settings, FPCT became the standard imaging modality for perioperative cochlear imaging benefitting from the aforementioned CBCT advantages [15–17]. A major drawback of this technique is the relatively longer acquisition time making it more susceptible to motion artefacts [11]. This challenge has been partially addressed through faster acquisitions [18, 19] and most recently through advancement of accelerated FPCT (Acc-FPCT) acquisition protocols available with the latest generation of C-arm angiography systems. High rotation speed of the C-arm allowed to shorten the scan time by 65% [20].

This cadaveric-based study aims to assess the potential role of these new techniques in preoperative temporal bone imaging and to evaluate any changes in radiation exposure. Specifically, we investigate the diagnostic performance of Acc-FPCT protocols compared to a latest-generation HR-MSCT in visualizing critical anatomical landmarks of the middle and inner ear.

## Methods

### Cadaveric specimens

For the purpose of this study and ensuing projects we obtained five intact cadaveric whole-head specimens, pre-designated for the institutional body donation program of the Institute of Anatomy of the Otto-von-Guericke University Magdeburg. All donors of the present study have individually given written informed consent, which explicitly included the post-mortem use of the body for scientific research. The requirement for ethics approval was deemed unnecessary according to relevant regulations for this research project by the institutional ethics committee.

All specimens had intact inner ear structures. Except for one specimen with partial mastoidectomy, all other heads had intact mastoids and ossicular chains. Due to data protection regulations in Germany, the Institute of Anatomy has no access to the patient data of the body donors. Medical histories of the cadavers were not disclosed.

### MSCT imaging

MSCT imaging was performed using a latest generation CT scanner (Somatom X.Cite, Siemens Healthineers, Forchheim, Germany) [21]. The specimens were positioned in the gantry isocenter in supine orientation.

A high-dose temporal bone protocol was applied with a higher tube current than that used in our clinical setting with the purpose of obtaining highest image quality possible. The protocol parameters were as follow: Voltage,120 kV; current, 384 mA; acquisition field of view (FOV): 25 × 5 cm; single collimation width: 0.6 mm; pitch factor 0.85, slice thickness 0.5 mm, scan duration 0.9 s.

Standardized reconstructions using a bone kernel (kernel J70h) were acquired with 0.5 mm slice thickness with a predetermined window level (WL) at 560 HU and window width (WW) of 3620 HU. Changing the windowing setting was allowed during rating.

### FPCT imaging

A total of six FPCT protocols were acquired on the specimens using an ICONO biplane angiography system [22] (Siemens Healthineers, Forchheim, Germany):

Two 8 s circular FPCT protocols (cFPCT) using 200° rotation angle and 496 frames with tube voltage of 70 kV and 109 kV respectively. Two sinusoidal FPCT (sFPCT) protocols with a duration of 7 s and 9 s respectively, using a sinusoidal curve rotation around the isocenter resulting in 10% higher angular coverage (220° rotation angle) and 50 additional frames in comparison to conventional cFPCT [23].

Two high resolution FPCT (HR-FPCT) protocols with limited field of view, “head micro”, as coined by the manufacturer, with scan duration of 7 s and 14 s respectively. The FOV is limited by default to 10 × 5 cm. Hence, the temporal bones of each cadaveric head were imaged separately. The protocols parameters are summarized in Table 1.

**Table 1:** Technical parameters of the tested scanning protocols

	cFPCT 8 s 70 kV	cFPCT 8 s 109 kV	sFPCT 7 s	sFPCT 9 s	HR-FPCT 7 s	HR-FPCT 14 s
scan duration (sec)	8.01	8.01	6.67	8.81	6.88	13.82
Slice thickness (mm)	0.15	0.15	0.15	0.15	0.15	0.15
Rotation angle	200°	200°	220°	220°	200°	200°
Tube voltage (kV)	70	109	115	115	98	100
Tube current (mAs)	95	105	182	197	231	208
Frame rate (F/s)	68	68	90	68	40	40
Matrix	512 × 512	512 × 512	512 × 512	512 × 512	512 × 512	512 × 512

Abbreviations: cFPCT: Circular flat panel computed tomography. sFPCT: Sinusoidal flat panel computed tomography. HR-FPCT: High-resolution flat panel computed tomography

FPCT data were reconstructed using the Artis Icono post-processing platform (Siemens Healthineers, Forchheim, Germany). Standardized reconstructions of all protocols using kernel type “HU” and image characteristic “sharp” were acquired with 512 × 512 matrices and isotropic voxel size of 0.15 mm. Changing the windowing setting was allowed during rating.

**Fig. 1:**
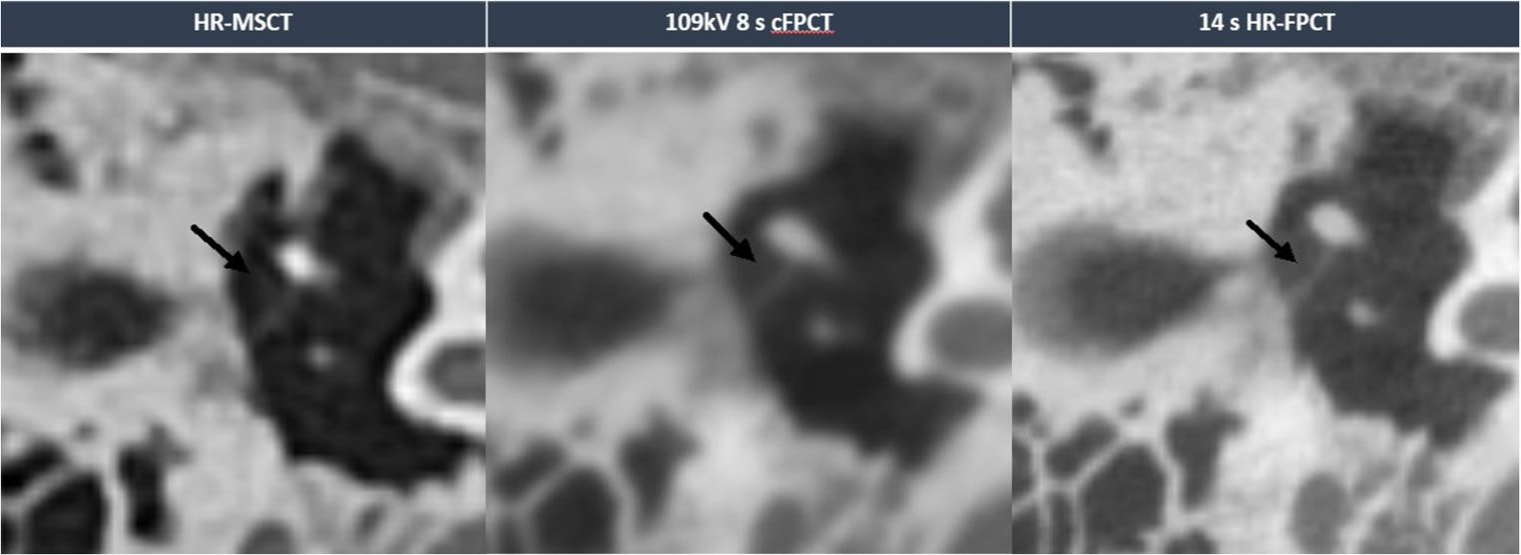
Illustrative example of different protocols in the visualization of the tympanic cavity. The HR-FPCT protocol allowed for superior visualization of the tympanic segment of chorda tympani (black arrow) in comparison to HR-MSCT and non-HR-FPCT

**Fig. 2:**
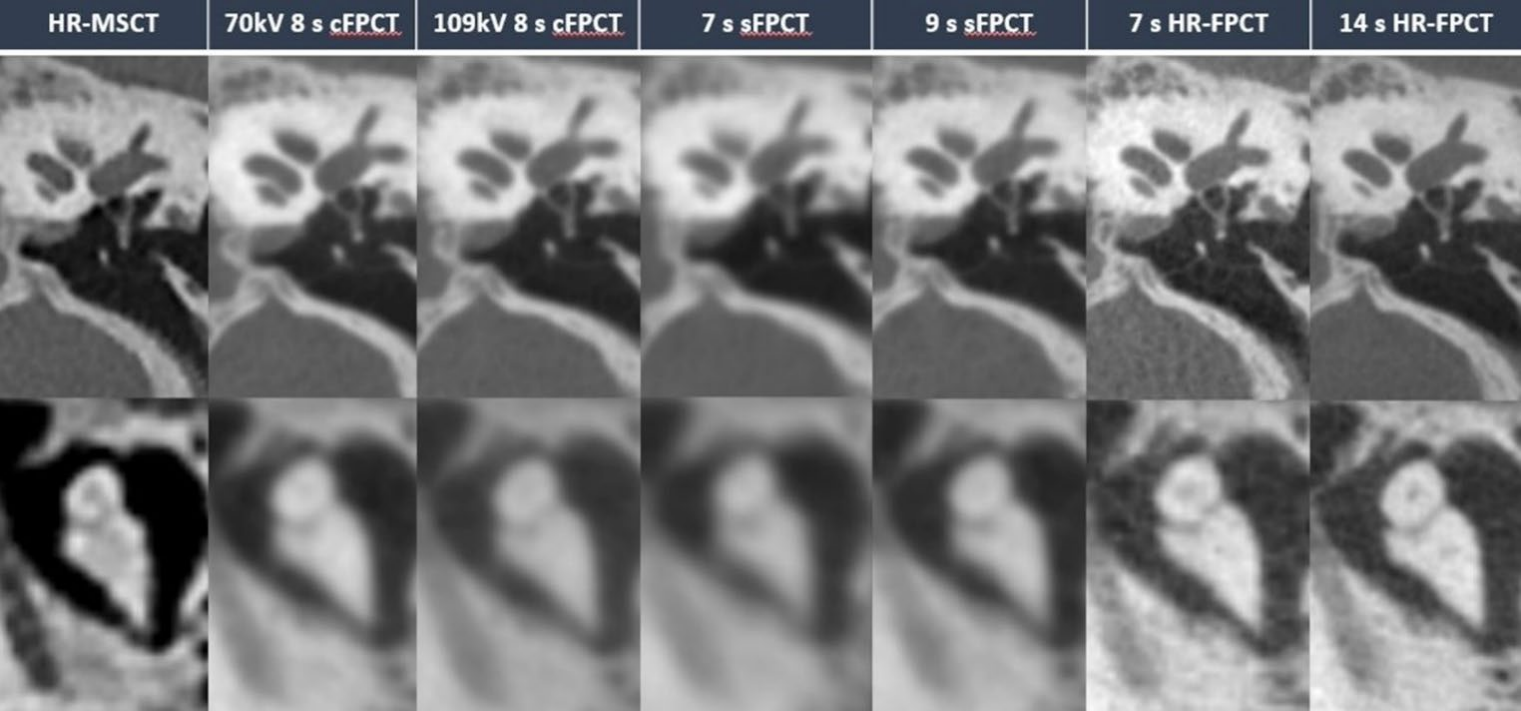
Illustrative example of the different protocols in the visualization of middle ear landmarks, stapes (top row) and incudomalleolar joint (lower row). Only HR-FPCT protocols allowed for superior visualization of the stapedial landmarks and obvious delineation of incudostapedial joint

**Fig. 3:**
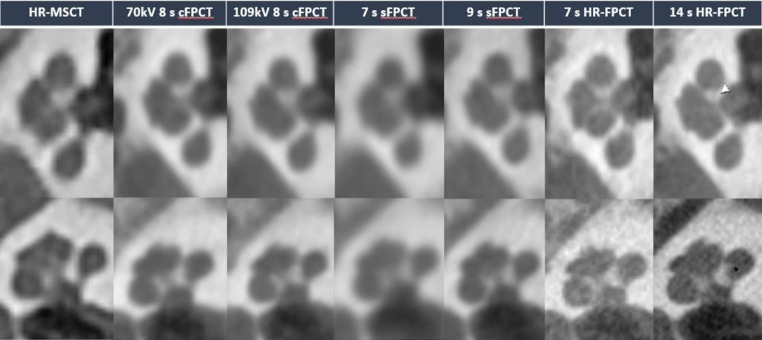
Illustrative example of the different protocols in the visualization of cochlear landmarks, parasagittal view (top row) and axial view (lower row). HR-FPCT protocols allowed for superior visualization. Minor structures such as longitudinal canals of the modiolus (white arrowhead) and spiral lamina (black arrowhead) were occasionally visualized in the 14 s HR-FPCT protocol

Illustrative examples of HR-MSCT and the different Acc-FPCT protocols are provided in Figs. 1, 2 and 3. 

### Imaging evaluation

Three neuroradiologists with extensive experience in temporal bone imaging assessed the image quality of each FPCT protocol for the visualization of 31 middle and inner ear anatomical landmarks including a checklist of structures usually evaluated in preoperative assessment for cochlear implantation and middle ear surgery.

The visibility of individual structures in all FPCT protocols were rated using a 5-point Likert scale questionnaire. HR-MSCT was used as the reference standard against which the different FPCT protocols were compared. As such, HR-MSCT was assigned a fixed score of 3 in our Likert-scale questionnaires. The FPCT protocols were then scored relative to HR-MSCT, with scores ranging from 1 to 5 (5 = superior with improved visibility, 4 = slightly superior, 3 = equivalent to HR-MSCT, 2 = slightly inferior, 1 = inferior with degraded visibility). The following criteria were used to standardize evaluation and reduce subjectivity in rating when applicable: Sharpness of structure boundaries, discernibility of anatomical relationship to neighboring structures and ability to accurately measure the structure. Although the three raters were blinded to the protocol names, the protocols were in part inherently recognizable.

A commercially available PACS viewer (Infinitt PACS, Infinitt Healthcare, Seoul, South Korea) was used to analyze all datasets.

### Dosimetry

Radiation dose parameters were recorded in dosimetry reports. For FPCT protocols, computed tomography dose index (CTDIvol) values in mGy and Dose length products (DLP) values in mGy.cm were extracted from the individual reports. The doses for FPCT were reported per cadaveric head, representing the combined radiation exposure from scanning both temporal bones separately.

For MSCT the DLP values were calculated by multiplying CTDIvol to scan length.

### Statistical analysis

Statistical analysis was performed using Python (3.9.18).

The statistical differences of cumulative protocol scores and scores for individual structures were compared between each FPCT protocol and MSCT using Wilkoxon Signed-Rank test.

Interrater reliability was assessed using intra-class correlation (ICC). A two-way random effect ICC form based on absolute agreement among multiple raters (ICC 2,k) was calculated [24].

## Results

### Protocol rating

Overall, only the two HR-FPCT protocols were rated as superior to HR-MSCT (*p* < 0.001) with cumulative score (mean sum) of “123.01” for the 14 s protocol and “115.9” for the 7 s protocol. HR-FPCT protocols consistently outperformed HR-MSCT when comparing individual landmarks except for spiral lamina where they yielded similar results.

When comparing the two HR-FPCT protocols to one another, the image quality score was slightly higher in the 14 s HR-FPCT than in the 7 s HR-FPCT with overall mean values of 3.97 (± 0.56) and 3.74 (± 0.51) respectively. The difference was not statistically significant (*p* = 0.25). The performance of the 14 s protocol was noticeably better in the visualization of ossicular chain landmarks with mean scores of 4.32 (± 0.45) and 3.92 (± 0.44), however without reaching statistical significance (*p* = 0.14).

In contrast, the four non HR-FPCT protocols were rated inferior to HR-MSCT (*p* < 0.001) with cumulative scores ranging from “57.76” for the 7 s sFPCT to “68.05” for the 109 kV 8 s cFPCT. In the evaluation of individual structures, they consistently underperformed HR-MSCT. The image quality scores of the latter protocols were particularly worse for bony modiolus, spiral lamina, saccular and posterior ampullary nerve canals, and ossicular chain landmarks.

**Fig. 4:**
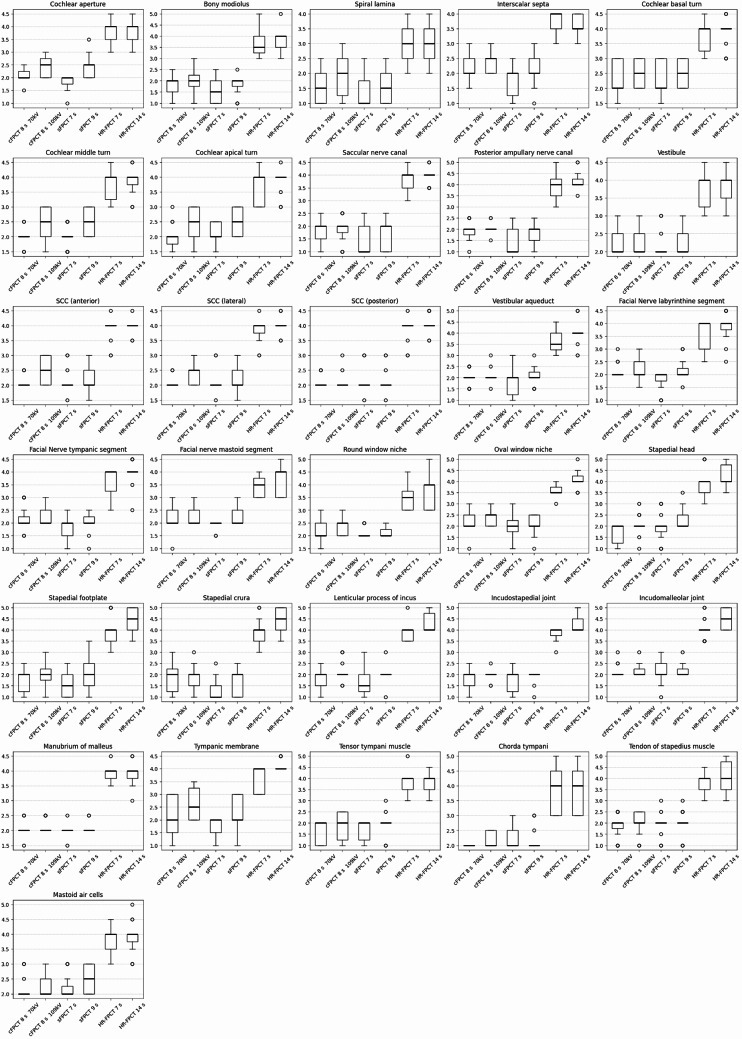
Graphic representation of rating results of middle and inner ear structures. Interquartile range (IQR) Q1 to Q3; whiskers: +/- 1.5 IQR). Abbreviations: SCC: Semicircular canals

A graphic representation of rating results for all structures is provided in Fig. 4.

For a comprehensive overview of image quality results, please refer to Table 2.

**Table 2:** Overview of image quality results

	cFPCT 8 s 70 kV	cFPCT 8 s 109 kV	sFPCT 7 s	sFPCT 9 s	HR-FPCT 7 s	HR-FPCT 14 s	cFPCT 8 s 70 kV/ MSCT	cFPCT 8 s 109 kV/ MSCT	sFPCT 7 s/ MSCT	sFPCT 9 s/ MSCT	HR-FPCT 7 s/ MSCT	HR-FPCT 14 s/ MSCT
Bony modiulus	1.73 ± 0.44	1.97 ± 0.53	1.5 ± 0.48	1.8 ± 0.44	3.7 ± 0.57	3.73 ± 0.51	< 0.0001*	< 0.0001*	< 0.0001*	< 0.0001*	0.0004	0.0001*
Chorda tympani	2.0 ± 0.0	2.2 ± 0.24	2.23 ± 0.36	2.17 ± 0.35	3.9 ± 0.69	3.93 ± 0.77	< 0.0001*	< 0.0001*	< 0.0001*	< 0.0001*	0.0002*	0.0005*
Cochlear aperture	2.1 ± 0.27	2.43 ± 0.4	1.83 ± 0.3	2.4 ± 0.45	3.73 ± 0.4	3.73 ± 0.4	< 0.0001*	0.0001*	< 0.0001*	0.0002*	< 0.0001*	< 0.0001*
Cochlear apical turn	2.0 ± 0.41	2.5 ± 0.52	2.1 ± 0.37	2.47 ± 0.46	3.7 ± 0.51	3.87 ± 0.39	< 0.0001*	0.0028*	< 0.0001*	0.0007*	0.0002*	< 0.0001*
Cochlear basal turn	2.33 ± 0.57	2.53 ± 0.46	2.37 ± 0.5	2.5 ± 0.45	3.73 ± 0.48	3.9 ± 0.42	0.0006*	0.0021*	0.0003*	0.0009*	0.0001*	< 0.0001*
Cochlear middle turn	2.0 ± 0.26	2.53 ± 0.5	2.0 ± 0.32	2.5 ± 0.45	3.73 ± 0.48	3.87 ± 0.43	< 0.0001*	0.0035*	< 0.0001*	0.0009*	0.0001*	< 0.0001*
Facial Nerve, labyrinthine Segment	2.13 ± 0.29	2.27 ± 0.44	1.8 ± 0.36	2.17 ± 0.39	3.57 ± 0.57	3.87 ± 0.53	< 0.0001*	< 0.0001*	< 0.0001*	< 0.0001*	0.0024*	< 0.0001*
Facial Nerve, mastoid Segment	2.17 ± 0.43	2.27 ± 0.4	1.93 ± 0.17	2.27 ± 0.31	3.47 ± 0.39	3.6 ± 0.52	< 0.0001*	< 0.0001*	< 0.0001*	< 0.0001*	0.0005*	0.0007*
Facial Nerve, tympanic Segment	2.13 ± 0.43	2.27 ± 0.36	1.77 ± 0.48	2.03 ± 0.39	3.63 ± 0.5	3.97 ± 0.46	< 0.0001*	< 0.0001*	< 0.0001*	< 0.0001*	0.0003*	< 0.0001*
Incudomalleolar joint	2.13 ± 0.29	2.17 ± 0.3	2.13 ± 0.5	2.17 ± 0.3	4.07 ± 0.44	4.53 ± 0.43	< 0.0001*	< 0.0001*	< 0.0001*	< 0.0001*	< 0.0001*	< 0.0001*
Incudostapedial joint	1.73 ± 0.4	1.97 ± 0.22	1.73 ± 0.51	1.9 ± 0.27	3.83 ± 0.3	4.33 ± 0.39	< 0.0001*	< 0.0001*	< 0.0001*	< 0.0001*	< 0.0001*	< 0.0001*
Incus, lenticular process	1.8 ± 0.36	2.1 ± 0.42	1.67 ± 0.54	2.0 ± 0.37	3.9 ± 0.37	4.37 ± 0.43	< 0.0001*	< 0.0001*	< 0.0001*	< 0.0001*	< 0.0001*	< 0.0001*
Interscalar septa	2.23 ± 0.36	2.37 ± 0.39	1.7 ± 0.48	2.13 ± 0.5	3.7 ± 0.4	3.63 ± 0.39	< 0.0001*	< 0.0001*	< 0.0001*	< 0.0001*	< 0.0001*	< 0.0001*
Spiral lamina	1.53 ± 0.53	1.93 ± 0.73	1.4 ± 0.55	1.63 ± 0.59	3.07 ± 0.57	2.93 ± 0.7	< 0.0001*	0.0001*	< 0.0001*	< 0.0001*	0.6702	0.7284
Malleus, manubrium	2.03 ± 0.22	2.1 ± 0.2	2.0 ± 0.18	2.07 ± 0.17	3.9 ± 0.27	3.9 ± 0.37	< 0.0001*	< 0.0001*	< 0.0001*	< 0.0001*	< 0.0001*	< 0.0001*
Mastoid air cells	2.17 ± 0.35	2.33 ± 0.39	2.2 ± 0.36	2.47 ± 0.46	3.83 ± 0.35	3.93 ± 0.51	< 0.0001*	< 0.0001*	< 0.0001*	0.0007*	< 0.0001*	< 0.0001*
Oval window	2.17 ± 0.43	2.33 ± 0.35	1.93 ± 0.57	2.07 ± 0.4	3.6 ± 0.27	4.07 ± 0.4	< 0.0001*	< 0.0001*	< 0.0001*	< 0.0001*	< 0.0001*	< 0.0001*
Posterior ampullary nerve canal	1.93 ± 0.4	2.03 ± 0.22	1.5 ± 0.58	1.8 ± 0.48	3.93 ± 0.6	4.17 ± 0.39	< 0.0001*	< 0.0001*	< 0.0001*	< 0.0001*	< 0.0001*	< 0.0001*
Round window niche	2.17 ± 0.39	2.4 ± 0.37	2.07 ± 0.17	2.13 ± 0.22	3.43 ± 0.48	3.77 ± 0.68	< 0.0001*	< 0.0001*	< 0.0001*	< 0.0001*	0.0044*	0.0009*
Saccular nerve canal	1.83 ± 0.51	1.87 ± 0.43	1.5 ± 0.58	1.7 ± 0.54	3.8 ± 0.4	3.97 ± 0.29	< 0.0001*	< 0.0001*	< 0.0001*	< 0.0001*	< 0.0001*	< 0.0001*
Semicircular canal (anterior SCC)	2.07 ± 0.17	2.43 ± 0.44	2.13 ± 0.39	2.2 ± 0.4	3.87 ± 0.39	3.97 ± 0.22	< 0.0001*	0.0003*	< 0.0001*	< 0.0001*	< 0.0001*	< 0.0001*
Semicircular canal (lateral SCC)	2.07 ± 0.17	2.37 ± 0.34	2.1 ± 0.37	2.27 ± 0.4	3.83 ± 0.39	4.0 ± 0.26	< 0.0001*	< 0.0001*	< 0.0001*	< 0.0001*	< 0.0001*	< 0.0001*
Semicircular canal (posterior SCC)	2.07 ± 0.17	2.1 ± 0.27	2.1 ± 0.37	2.13 ± 0.39	3.87 ± 0.39	4.03 ± 0.29	< 0.0001*	< 0.0001*	< 0.0001*	< 0.0001*	< 0.0001*	< 0.0001*
Stapes, Crura	1.87 ± 0.67	1.87 ± 0.56	1.37 ± 0.46	1.67 ± 0.51	3.93 ± 0.54	4.43 ± 0.51	< 0.0001*	< 0.0001*	< 0.0001*	< 0.0001*	< 0.0001*	< 0.0001*
Stapes, footplate	1.73 ± 0.51	1.97 ± 0.62	1.53 ± 0.5	2.03 ± 0.76	4.0 ± 0.58	4.47 ± 0.46	< 0.0001*	< 0.0001*	< 0.0001*	0.0003*	< 0.0001*	< 0.0001*
Stapes, head	1.67 ± 0.43	2.03 ± 0.43	1.93 ± 0.6	2.3 ± 0.51	3.87 ± 0.56	4.23 ± 0.54	< 0.0001*	< 0.0001*	< 0.0001*	0.0002*	< 0.0001*	< 0.0001*
Tendon of stapedius muscle	1.87 ± 0.5	2.0 ± 0.48	1.93 ± 0.48	2.0 ± 0.48	3.73 ± 0.48	4.07 ± 0.7	< 0.0001*	< 0.0001*	< 0.0001*	< 0.0001*	0.0001*	0.0001*
Tensor tympani muscle	1.63 ± 0.46	1.87 ± 0.59	1.67 ± 0.43	1.97 ± 0.46	3.8 ± 0.48	3.87 ± 0.43	< 0.0001*	< 0.0001*	< 0.0001*	< 0.0001*	< 0.0001*	< 0.0001*
Tympanic membrane	2.03 ± 0.76	2.57 ± 0.63	1.7 ± 0.4	2.2 ± 0.75	3.53 ± 0.5	4.1 ± 0.2	0.0003*	0.0219*	< 0.0001*	0.0013*	0.0013*	< 0.0001*
Vestibular aqueduct	2.0 ± 0.32	2.07 ± 0.31	1.77 ± 0.57	2.07 ± 0.4	3.6 ± 0.45	3.97 ± 0.53	< 0.0001*	< 0.0001*	< 0.0001*	< 0.0001*	0.0002*	< 0.0001*
Vestibule	2.2 ± 0.31	2.2 ± 0.31	2.17 ± 0.35	2.33 ± 0.39	3.7 ± 0.48	3.8 ± 0.36	< 0.0001*	< 0.0001*	< 0.0001*	< 0.0001*	0.0001*	< 0.0001*

Rating results of the 3 independent raters for *n* = 10 temporal bones, mean values ± standard deviation, with statistical significance indicated by * (*p* < 0.05)

Abbreviations: cFPCT: Circular flat panel computed tomography. sFPCT: Sinusoidal flat panel computed tomography. HR-FPCT: High-resolution flat panel computed tomography. SCC: Semicircular canals

### Inter-rater reliability

The interrater reliability was excellent ICC (2,k) = 0.925; CI [0.92–0.93].

### Dosimetry

The HR-MSCT yielded the highest dose parameters with a mean DLP of 324 mGy.cm (298.5-351.6). The 70 kV 8 s cFPCT had the lowest mean DLP value among the non HR-FPCT protocols at 158 mGy.cm, whereas the 14 s HR-FPCT had the highest at 288 mGy.cm.

Due to their increased rotation angle, the sFPCT protocols with higher mean DLP values at 232 and 244 mGy.cm respectively, even exceeding that of 7 s HR-FPCT protocol (226 mGy.cm).

Mean DLP values in mGy.cm of all FPCT protocols and HR-MSCT were summarized in Table 3.

**Table 3:** Dose parameters of HR-MSCT and FPCT protocols

	CTDIvol (mGy)	DLP (mGy.cm)
HR-MSCT	64.8	324
cFPCT 8 s 70kv	31.6	158
cFPCT 8 s 109kv	36.6	183
sFPCT 7 s	46.4	232
sFPCT 9 s	48.9	244
HR-FPCT 7 s	45.2	226
HR-FPCT 14 s	57.2	288

Abbreviations: cFPCT: Circular flat panel computed tomography, sFPCT: Sinusoidal flat panel computed tomography, HR-FPCT: High-resolution flat panel computed tomography, HR-MSCT: High-resolution multi-slice computed tomography, CTDIvol: CT dose index. DLP: Dose length product

## Discussion

In the last two decades multiple studies demonstrated that CBCT is at least comparable to MSCT in the assessment of anatomical landmarks of middle and inner ear [1, 25–28]. Although these studies predominantly used dedicated digital volume tomography (DVT) CBCT systems, the utility of using multipurpose commercially available C-arm mounted FPCT systems was established [16, 17]. These findings were further supported by two comparative studies, showing that FPCT provided superior image quality compared to MSCT in visualization of temporal bone and skull base structures, even with faster 10 s FPCT acquisition protocols [18, 19]. Our findings for the HR-FPCT protocols align with those of previous studies, demonstrating superior image quality compared to HR-MSCT across all investigated structures, with the exception of spiral lamina visualization.

### Use of Acc-FPCT protocols in temporal bone imaging

The utility of Acc-FPCT protocols available with the latest generation of C-arm angiography systems was first investigated by Eisenhut et al. [20]. The group observed superior image quality in all Acc-FPCT protocols investigated compared to a 128-row MSCT scanner (Somatom Definition AS +, Siemens Healthcare GmbH, Erlangen, Germany) using the Stuffert et al. criteria [17] as a benchmark. In our study, only HR-FPCT protocols were shown to be superior to HR-MSCT. However, HR-MSCT outperformed all other FPCT protocols with superior image quality for all assessed structures. This may be explainable by the higher image quality of MSCT images conducted on a latest generation scanner (Somatom X.cite, Siemens Healthcare GmbH, Erlangen, Germany) with adjusted scan parameters aimed at maximizing image quality.

Not only did the HR-FPCT protocols provide superior image quality, but they also required only 70% and 89% of the dose in the 7 s and 14 s scans, respectively, compared to HR-MSCT.

When comparing the two HR-FPCT protocols to one another, we found no statistically significant differences in image quality, which advocates the use of the shorter protocol requiring 22% less dose.

Furthermore, we found no benefit in using sinusoidal rotation protocols (sFPCT), which showed relatively degraded image quality at the cost of increased radiation dose. These protocols were reported to be useful in reducing beam-hardening artefacts in stroke imaging [23, 29, 30], however, to our best knowledge, they haven’t been investigated in temporal bone imaging yet.

### Limitations

Beside inherent limitations of cadaveric bone imaging studies such as altered bone mineral density and changes in tissue consistency, the main limitation to the applicability of our findings in in-vivo setting is the lack of motion-related artefacts in cadaveric imaging. Although the acquisition times of Acc-FPCT protocols were shortened to 7 s, the vulnerability to motion-related artefacts is still higher than in MSCT, which involves much faster acquisition time of less than one second. Such artefacts could degrade the image quality of FPCT favoring MSCT in clinical setting. Hence, further in-vivo studies are warranted to validate the utility of Acc-FPCT protocols in temporal bone imaging.

Methodological limitations to our study include the reliance on CTDIvol and DLP values for dose comparisons as no phantom measurements were applied.

Second, HR-MSCT acquisitions using elevated tube currents aimed at maximizing image quality resulted in higher radiation dose. Although the CTDIvol and DLP values of HR-MSCT remained within the upper margin of the diagnostic reference levels, they exceeded those applied in temporal bone scans in our clinical routine by a factor of 1.4. The dose values of the investigated Acc-FPCT protocols (except for the 14 s HR-FPCT) were still lower when compared to those “real-world” MSCT dose values. We did not conduct a comparison to a low-dose MSCT protocol. A study by Kofler et al. reported that low-dose MSCT protocols, with CTDIvol as low as 7.66 mGy, may be sufficient for visualization of temporal bone landmarks [31]. Such protocols could offer significant advantages when scanning children, given their considerably lower radiation doses.

Third, the difference in size of FOV between HR-MSCT and FPCT. Due to the inherently limited acquisition volume in FPCT, a concentrated field of view (FOV) is selected to image each temporal bone separately. In contrast, HR-MSCT employs a wider FOV that encompasses both temporal bones simultaneously, which partially compromises the comparability of dosimetry between the two modalities. To account for this discrepancy in acquisition volumes between the two modalities, the FPCT doses were reported per cadaveric head, representing the combined radiation exposure from scanning both temporal bones separately. This adjustment improves the comparability with MSCT, though we acknowledge a residual discrepancy due to the difference between the complete cross-sectional acquisition volume in MSCT and the combined acquisition volumes of the bilateral temporal bones in FPCT. The same methodological issue was encountered in similar studies comparing CBCT to MSCT [1, 11, 25].

## Conclusion

The findings of our cadaveric-based study further highlight the utility of Acc-FPCT available with latest generation of C-arm angiography systems in preoperative temporal bone assessment. Certain HR-FPCT protocols demonstrated superior image quality without increasing radiation exposure compared to a non-dose-optimized HR-MSCT. Due to their fast acquisition times, they could mitigate motion artefacts usually encountered in CBCT imaging. While the results suggest potential advantages, further in-vivo studies are warranted to fully validate their clinical potential. As the latest generation of C-arm mounted angiography systems becomes more accessible, there is potential for increased reliance on this technology in the preoperative temporal bone imaging workup, potentially reshaping the workflow and enhancing the versatility of imaging options available to clinicians.

Looking ahead, as we move into the era of photon counting CT, it will be crucial to benchmark FPCT against this emerging technology, which has shown promising results in temporal bone imaging.

## Data Availability

No datasets were generated or analysed during the current study.

## Abbreviations

Acc-FPCT: Accelerated flat panel computed tomography
CBCT: Cone beam computed tomography
CI: Confidence interval
cFPCT: Circular flat panel computed tomography
CT: Computed tomography
CTDIvol: Computed tomography dose index
DLP: Dose length product
DVT: Digital volume tomography
DWI: Diffusion-weighted imaging
FOV: Field of view
HR-MSCT: High-resolution multisclice computed tomography
HU: Hounsfield unit
ICC: Intraclass correlation coefficient
MSCT: Multi-slice computed tomography
PACS: Picture archiving and communication system
sFPCT: Sinusoidal flat panel computed tomography
